# Supplementary material for: The interactome of intact mitochondria by cross-linking mass spectrometry provides evidence for coexisting respiratory supercomplexes
Source: Mol Cell Proteomics. 2017 Dec 8;17(2):216–32. doi: 10.1074/mcp.RA117.000470 (PMC5795388; doi:10.1074/mcp.RA117.000470)
Supplement: Supplemental Data [file supp_RA117.000470_133922_0_supp_23978_fzffwf.zip › spectra_annotation/mito_spectra_annotation/166-2-5-1-6-1.pdf]

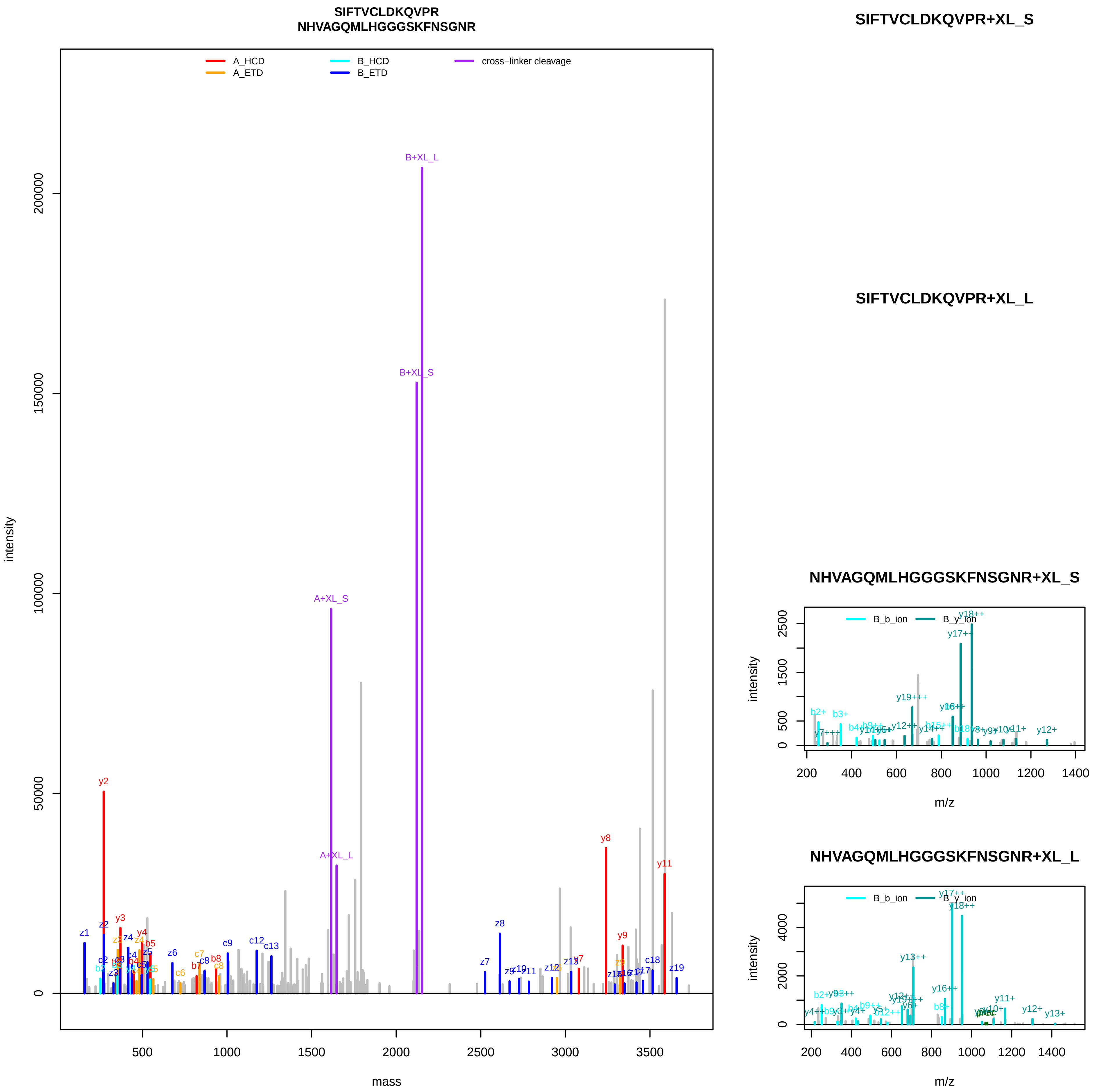

SIFTVCLDKQVPR
NHVAGQMLHGGGSKFNSGNR
A_HCD
A_ETD
B_HCD
B_ETD
cross−linker cleavage
B+XL_L
B+XL_S
A+XL_S
A+XL_L
y2
y3
y4
b5
y8
y9
y11
y7
b7
b8
z1
z2
z4
z5
z6
z7
z8
z11
z12
z13
z19
c8
c9
c12
c13
c18
c7
c6
intensity
mass
0
50000
100000
150000
200000
500
1000
1500
2000
2500
3000
3500
SIFTVCLDKQVPR+XL_S
SIFTVCLDKQVPR+XL_L
NHVAGQMLHGGGSKFNSGNR+XL_S
B_b_ion
B_y_ion
y18++
y17++
y19+++
y16++
y12++
y12+
b2+
b3+
m/z
0
500
1500
2500
200
400
600
800
1000
1200
1400
NHVAGQMLHGGGSKFNSGNR+XL_L
y13++
y11+
y12+
y13+
b2+
0
2000
4000
